# Bing-Neel Syndrome: A Missed Opportunity

**DOI:** 10.7759/cureus.79144

**Published:** 2025-02-17

**Authors:** David R Drysdale, Mark E Gartner, Ryan A Smith, Bradley J Atoa, Aaron D Fleetwood, Tyson J Sjulin, Michael B Osswald

**Affiliations:** 1 Department of Medicine, Division of Internal Medicine, Brooke Army Medical Center, San Antonio, USA; 2 Department of Medicine, Division of Pulmonary and Critical Care Medicine, Brooke Army Medical Center, San Antonio, USA; 3 Department of Pathology, Brooke Army Medical Center, San Antonio, USA; 4 Department of Radiology, Brooke Army Medical Center, San Antonio, USA; 5 Department of Medicine, Division of Hematology and Oncology, Brooke Army Medical Center, San Antonio, USA

**Keywords:** bing-neel syndrome, bruton tyrosine kinase inhibitor, ibrutinib, lymphoplasmacytic lymphoma, waldenstrom macroglobinemia

## Abstract

Bing-Neel syndrome (BNS) is a rare complication of Waldenström macroglobulinemia, characterized by lymphoplasmacytic lymphoma infiltration of the CNS. We present a pathologically confirmed case of BNS in a patient who was initially misdiagnosed with COVID-19 pneumonia and critical illness polyneuropathy. This case underscores the diagnostic challenges associated with BNS and highlights the importance of early recognition.

## Introduction

Bing-Neel syndrome (BNS) is a rare complication of Waldenström macroglobulinemia (WM), characterized by lymphoplasmacytic lymphoma (LPL) infiltration of the CNS [1-3]. While its true incidence remains unknown, it is estimated to occur in approximately 1% of WM cases [1-6]. The time from initial WM diagnosis to BNS onset varies widely, ranging from months to over a decade [1,3].

The diagnostic workup typically includes CSF analysis with flow cytometry, protein electrophoresis, immunofixation, and MRI of the brain and spinal cord [1-3]. Bruton tyrosine kinase (BTK) inhibitors, a class of small molecule inhibitors, are increasingly being used as a treatment option [2-4].

BNS presents with a broad spectrum of neurological symptoms, including motor and sensory deficits, gait disturbances, and cognitive impairment [1-3]. Due to its rarity, cognitive biases may contribute to diagnostic delays. Early recognition is crucial for improving survival rates, as uncertainty in diagnosis can postpone appropriate treatment.

This paper describes a subacute presentation of BNS that initially mimicked critical illness myopathy and led to prolonged mechanical ventilation. Additionally, it provides a detailed review of the radiographic and pathologic findings to enhance awareness of the diagnosis and management of this rare malignancy.

## Case presentation

A 77-year-old male with a past medical history of WM (IgM lambda-restricted, MYD88-mutated LPL) and associated chronic immune-mediated neuropathy, for which he received periodic IVIG transfusions, presented to the hospital with progressive dyspnea, symmetric lower extremity weakness, and bilateral facial droop while on vacation abroad. During his initial presentation, he underwent an evaluation for stroke, including a CT of the head and routine labs, which were unremarkable. Further neuroimaging was not completed at that time. He was also noted to have severe combined hypoxic and hypercapnic respiratory failure and tested positive for SARS-CoV-2 by PCR. Given the lack of a clearly identifiable alternative etiology, he was diagnosed with acute respiratory distress syndrome (ARDS) secondary to COVID-19 pneumonia and treated with corticosteroids, mechanical ventilation, and eventual tracheostomy placement after a prolonged ventilator course, which was complicated by ventilator-associated pneumonia. He was later compassionately transferred back to the United States for ongoing evaluation and management, including continued ventilator weaning.

Upon arrival, he was found to have severe septic shock requiring multiple vasopressors and evidence of multi-organ failure, including encephalopathy, pancytopenia, acute renal failure, acute liver injury, and persistent combined hypoxic and hypercapnic respiratory failure. Interestingly, his chest CT on arrival was unremarkable and specifically did not demonstrate typical findings of ARDS or COVID-19 pneumonia. A broader diagnostic evaluation was performed to investigate alternative etiologies of persistent encephalopathy. Given the high suspicion for hyperviscosity syndrome in the setting of WM, quantitative immunoglobulins, serum protein electrophoresis, serum viscosity measurement, and peripheral flow cytometry were obtained (Table 1). His IgM levels and M-spike were both below the pre-hospital baseline. Peripheral flow cytometric immunophenotyping demonstrated LPL, and serum MYD88 was detected by fluorescence in situ hybridization (FISH), consistent with his known diagnosis of WM.

**Table 1:** Key laboratory findings from serology and protein electrophoresis Studies were obtained both during and prior to hospitalization, with reference ranges provided. FISH, fluorescence in situ hybridization

Laboratory	During	Before	Reference range
IgG	317 mg/dL	Unavailable	700-1,600 mg/dL
IgA	<62 mg/dL	Unavailable	70-400 mg/dL
IgM	195 mg/dL	1,074 mg/dL	40-230 mg/dL
Viscosity	1.4 cP	1.7 cP	1.4-2.1 cP
Kappa light chain free	32.2 mg/L	13.1 mg/L	3.3-19.4 mg/L
Lambda light chain free	59.5 mg/L	147.5 mg/L	5.7-26.3 mg/L
Kappa/lambda ratio	0.54	0.09	0.26-1.65
Albumin fraction	2.42 g/dL	3.46 g/dL	3.63-4.72 g/dL
Alpha 1 globulin	0.35 g/dL	0.28 g/dL	0.23-0.38 g/dL
Alpha 2 globulin	0.67 g/dL	0.61 g/dL	0.55-0.95 g/dL
Beta globulin	0.26 g/dL	0.55 g/dL	0.64-1.05 g/dL
M-spike	0.32 mg/dL	1.16 g/dL	Not applicable
Gamma globulin	0.71 g/dL	2.39 g/dL	0.68-1.32 g/dL
Serum MYD88 FISH	Detected	Unavailable	Not applicable
Bone marrow MYD88 FISH	Not performed	Detected	Not applicable

**Figure 1:**
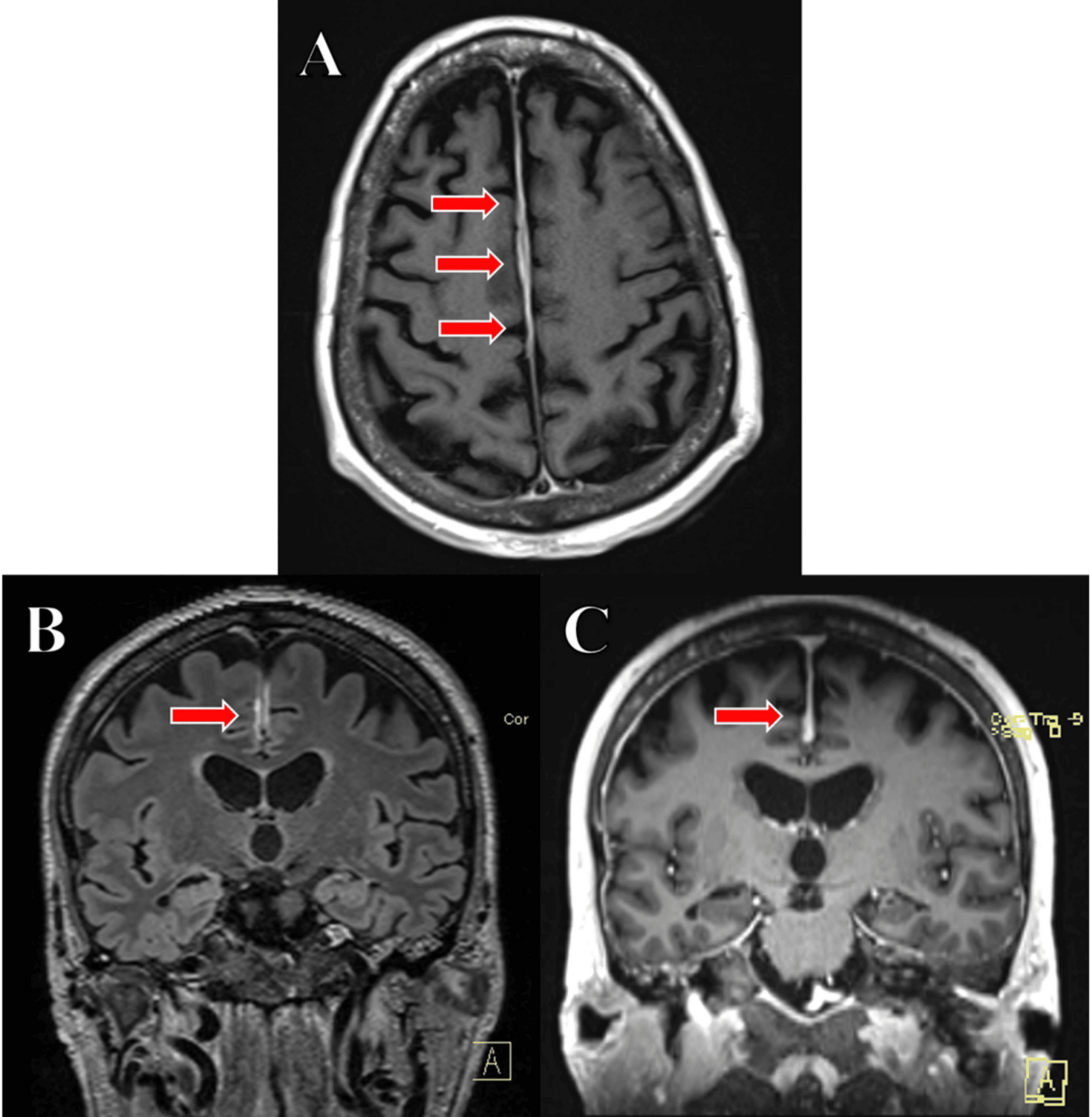
(A, top middle, red arrows) T1 post-contrast axial view showing smooth pachymeningeal enhancement along the bilateral cerebral convexities. (B, bottom left, red arrow) FLAIR coronal view revealing mild nodular dural enhancement along the mid falx. (C, bottom right, red arrow) Post-contrast coronal view demonstrating mild nodular dural enhancement along the mid falx.

An EEG was notable for diffuse slowing, consistent with metabolic encephalopathy, and negative for epileptiform discharges. Complete neuraxial MRI, both without and with contrast, demonstrated extensive bone marrow edema concerning an infiltrative process and pachymeningeal enhancement (Figure 1A-1C).

**Figure 2:**
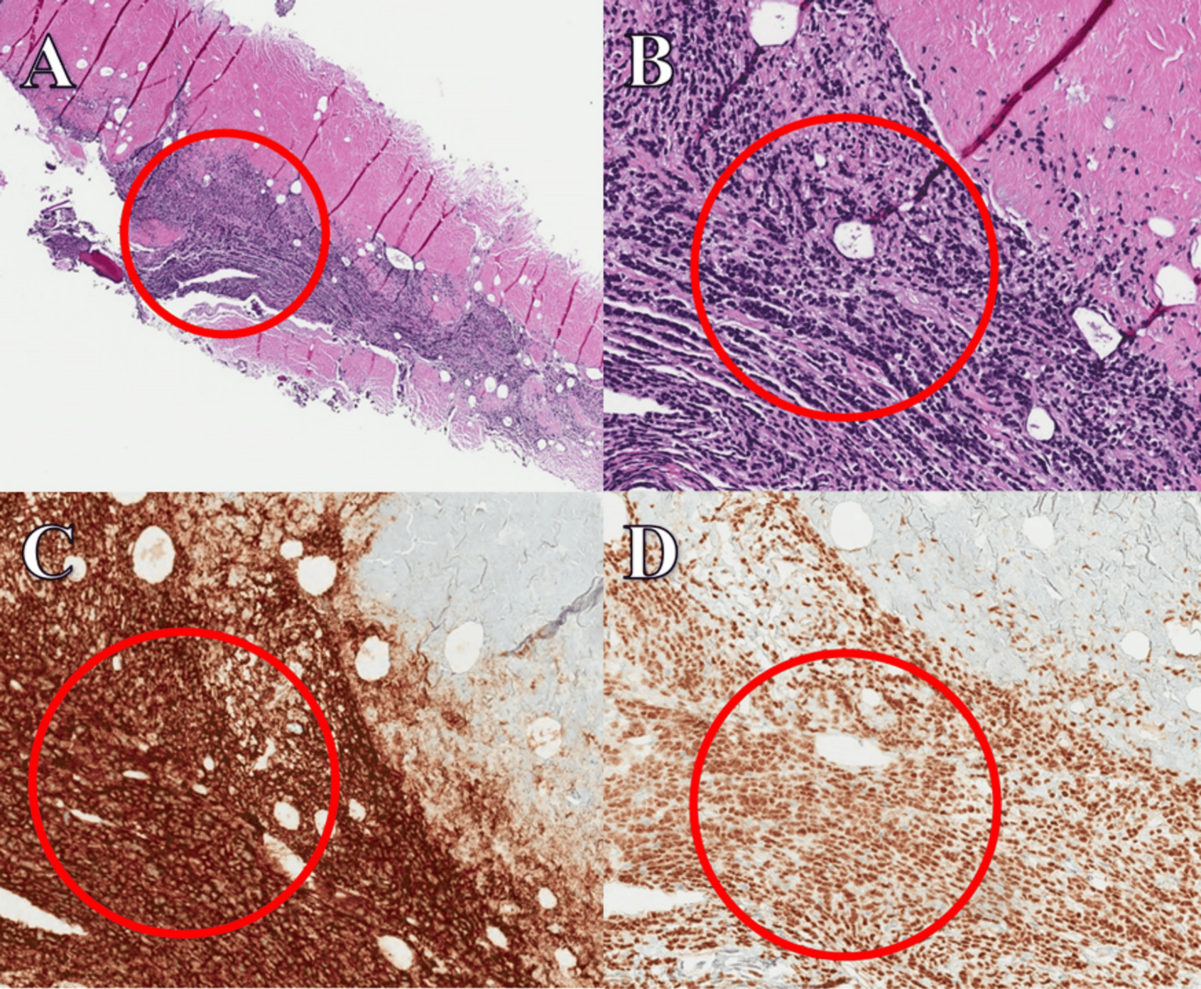
Hemispheric dura and falx demonstrating atypical LPL infiltrate on H&E staining at 200x. (A, top left, red circle) H&E stain showing LPL infiltrate at 40x. (B, top right, red circle) H&E stain showing LPL infiltrate at 200x. (C, bottom left, red circle) CD20-positive staining at 200x. (D, bottom right, red circle) PAX5-positive staining at 200x. LPL, lymphoplasmacytic lymphoma; PAX5, paired box 5 Image credit: Ryan A. Smith

Prior to the planned lumbar puncture under fluoroscopic guidance, the patient developed fulminant septic shock due to pansensitive Escherichia coli bacteremia, which ultimately led to his death. A limited CNS autopsy confirmed the presence of pachymeningeal and basal ganglia infiltration by atypical lymphoplasmacytes, consistent with BNS (Figure 2A-2D).

## Discussion

Radiography

MRI of the brain and spinal cord is crucial for diagnosing CNS lymphomas, including WM, and is highly recommended in the workup of BNS [1-12]. While histologic findings are diagnostic, imaging plays a key role in confirming the diagnosis, ruling out alternative conditions, and monitoring treatment response [1,3,4,10]. MRI findings can vary significantly between patients, but commonly include hypointense T1 signals with hyperintense T2 foci within the brain parenchyma, particularly in the subcortical or periventricular white matter. Medullary parenchymal involvement and optic nerve involvement have also been reported [9]. Brain diffusion-weighted imaging typically shows hyperintensities in cerebral and cerebellar lesions, which appear isointense on apparent diffusion coefficient maps, consistent with vasogenic edema [10]. Gadolinium contrast is often used for diagnosis, revealing enhancement in the medullary leptomeninges, cauda equina, and dura mater [9].

Two distinct radiographic forms of BNS have been described [1-4,8-12]. The first is the diffuse infiltrative form, which is associated with contrast enhancement of the leptomeninges and cauda equina, as seen in this case (Figure 1A-1C). The second is the tumoral form, characterized by unifocal or multifocal mass-like lesions in the subcortical regions of the brain. These lesions show hyperintensity on T2-weighted sequences, iso- or hypointensity on T1-weighted sequences, and contrast enhancement on T1-weighted gadolinium-enhanced sequences [10].

Pathology

The gold standard for diagnosing BNS involves the identification of LPL infiltration in the CNS, which can be demonstrated through histologic biopsy of the cerebrum or meninges, or CSF analysis, followed by flow cytometry, protein electrophoresis, and molecular diagnostics [1,2,5,6,10,11]. Malignant cells are typically monotypic B cells that express B cell antigens, including CD19, CD20, CD79a, CD27, and CD52 [3,4,10]. Monotypic plasma cells expressing CD138 and IgM may also be present. Additionally, mutations in the MYD88 gene, particularly the L265P mutation, are commonly found in lymphoid neoplasms and are often present in 93-97% of WM patients, including the patient in this case [1-4,6,7,9-11,13].

An autopsy plays a vital role in confirming the diagnosis when the premortem workup is incomplete, as seen in this case [11]. Figure 2A-2D illustrates the autopsy findings, revealing diffuse atypical LPL infiltration of the pachymeninges, with focal involvement in the basal ganglia. Immunohistochemistry (IHC) and FISH confirmed the LPL infiltration in the CNS, establishing the postmortem diagnosis of BNS.

The basal ganglia exhibited a lymphocytic perivascular infiltrate, positive for CD45 and CD20. Multiple sections of the pachymeninges, including the hemispheric dura and falx, demonstrated an atypical LPL infiltrate. The cells were diffusely positive for CD45, with a predominant subset of CD20-positive and paired box 5-positive B lymphocytes. IHC for CD138 and IgM, alongside FISH for kappa and lambda light chains, revealed rare, admixed lambda-predominant IgM plasma cells. These markers are characteristic of LPL.

Management

Any unexplained neurologic deficits in WM patients should prompt early evaluation with CNS MRI, CSF analysis, including flow cytometry, protein electrophoresis, and molecular diagnostics [1,2,5,10]. The median time from WM diagnosis to the development of BNS is approximately 8.9 years [9]. BNS has an overall survival (OS) rate of 71% at five years and 59% at 10 years [9]. Ibrutinib, a BTK inhibitor with good CNS penetration, should be considered as first-line therapy, as it confers an OS of 86% at five years [14]. However, C-X-C chemokine receptor type 4 mutations have been linked to resistance to ibrutinib in some cases [4]. Currently, there are no established prognostic factors for BNS, highlighting a critical area for future research.

## Conclusions

Neurologic symptoms in BNS often present vaguely and nonspecifically, including motor and sensory deficits, gait disturbances, and cognitive impairment. MRI with gadolinium contrast is highly recommended for diagnosing BNS and monitoring treatment response, typically revealing hypointense T1 signals and hyperintense T2 foci in the brain parenchyma, particularly with subcortical and periventricular involvement. Two radiographic forms of BNS are described: the diffuse infiltrative form and the tumoral form. Diagnosis is confirmed through histologic biopsy of the cerebrum or meninges or via lumbar puncture with CSF analysis showing LPL. MYD88 gene mutations (L265P) are commonly found in WM patients. An autopsy can confirm the diagnosis when the premortem workup is incomplete. Prompt CNS MRI and CSF analysis with flow cytometry are crucial for unexplained neurologic deficits in WM patients. The BTK inhibitor ibrutinib is considered first-line therapy due to its excellent CNS penetration and improved survival outcomes.

It is vital for physicians to approach each diagnostic evaluation systematically. Maintaining awareness of biases, staying updated on rare diseases, and collaborating with multidisciplinary teams can help shorten the “diagnostic odyssey,” ultimately improving patient outcomes.
